# Treatment as a moderator and executive function as a mediator of the effect of a mindfulness ecological momentary intervention for generalized anxiety disorder

**DOI:** 10.1017/S0033291724001958

**Published:** 2024-10

**Authors:** Nur Hani Zainal, Michelle G. Newman

**Affiliations:** 1 Department of Psychology, National University of Singapore, Kent Ridge, Singapore; 2 Department of Health Care Policy, Harvard Medical School, Boston, MA, USA; 3 Department of Psychology, The Pennsylvania State University, University Park, PA, USA

**Keywords:** change mechanism, ecological momentary intervention, executive functioning, generalized anxiety disorder, mediation, mindfulness, randomized controlled trial, worry

## Abstract

**Background:**

Theory and research indicated that executive functioning (EF) correlated with, preceded, and stemmed from worry in generalized anxiety disorder (GAD). The present secondary analysis (Zainal & Newman, 2023b) thus determined whether EF domains mediated the effect of a 14-day (5 prompts/day) mindfulness ecological momentary intervention (MEMI) against a self-monitoring control (SM) for GAD.

**Method:**

Participants (*N* = 110) diagnosed with GAD completed self-reported (Attentional Control Scale, GAD Questionnaire, Perseverative Cognitions Questionnaire) and performance-based tests (Letter-Number Sequencing, Stroop, Trail Making Test-B, Verbal Fluency) at baseline, post-treatment, and one-month follow-up (1MFU). Causal mediation analyses determined if pre-post changes in EF domains preceded and mediated the effect of MEMI against SM on pre-1MFU changes in GAD severity and trait repetitive negative thinking (RNT).

**Results:**

MEMI was more efficacious than SM in improving pre–post inhibition (*β* = −2.075, 95% [−3.388, −0.762], *p* = .002), working memory (*β* = 0.512, 95% [0.012, 1.011], *p* = .045), and set-shifting (*β* = −2.916, 95% [−5.142, −0.691], *p* = .010) but not verbal fluency and attentional control. Within groups, MEMI but not SM produced improvements in all examined pre–post EF outcomes except attentional control. Only pre–post improvements in inhibition mediated the effect of MEMI against SM on pre-1MFU reductions in GAD severity (*β* = −0.605, 95% [−1.357, −0.044], *p* = .030; proportion mediated = 7.1%) and trait RNT (*β* = −0.024, 95% [−0.054, −0.001], *p* = .040; proportion mediated = 7.4%). These patterns remained after conducting sensitivity analyses with non-linear mediator-outcome relations.

**Conclusions:**

Optimizing MEMI for GAD might entail specifically boosting inhibition plausibly by augmenting it with dialectical behavioral therapy, encouraging high-intensity physical exercises, and targeting negative emotional contrast avoidance.

*Executive function* (EF) is defined as a collection of advanced cognitive processes to manage purpose-guided actions and thoughts, encompassing abilities such as adaptability, memory, and planning (Friedman & Miyake, 2017). EF has been theorized to include *inhibitory control* (or *inhibition*; managing undesired or unhelpful behaviors), *shifting* (cognitively transitioning between various tasks), and *working memory* (WM; processing real-time inputs while attending to the task at hand; Miyake & Friedman, 2012) in related yet dissociable ways (Rodríguez-Nieto *et al*. 2022). *Verbal fluency* (VF) has been further recognized as a distinguishable EF component, connecting to more specialized language-based WM domains (Gustavson et al., 2019). Relatedly, *attentional control* (AC; subjective ability to maintain stable focus on the task at hand) has been posited to underpin EF, as it is closely related to meta-cognitive processes (Drigas & Karyotaki, 2017). Since problems with EF and AC are linked to various biopsychosocial issues (Lawson, Hook, & Farah, 2018; Woon, Farrer, Braman, Mabey, & Hedges, 2017; Yang, Shields, Guo, & Liu, 2018), developing efficacious interventions to enhance EF outcomes is crucial.

Mindfulness-based interventions (MBIs) have been theorized as potentially efficacious for EF by possibly enhancing resting-state functional connectivity between brain regions linked to EF (Taren et al., 2017). Further, intentionally focusing on the current moment without judgment and fully accepting emotions could aid in reorienting attention to the task and intended aims (Lutz et al., 2009), possibly enhancing AC and EF. Concordant with these propositions, a qualitative review observed that even brief MBIs could strengthen inhibition and WM EF domains (Zhou, Liu, & Deng, 2020). Extending that report, a meta-analysis of 111 randomized controlled trials (RCTs) found that MBIs *v.* active controls produced small-to-medium efficacy on executive attention, WM, inhibition, and set-shifting but did not differentially affect VF (Zainal & Newman, 2024b). Together, enhanced AC and EF might function as change mechanisms of the impact of MBIs on clinical outcomes, such as reductions in depression and anxiety symptoms (Blanck et al., 2018; Spijkerman, Pots, & Bohlmeijer, 2016).

Mediation research offers invaluable perspectives into proxy change mechanisms, frequently recognized as the central methodology in the exploration of how or why MBIs or other therapies work (Kazdin, 2007). It determines whether a mediator variable statistically elucidates the link between treatment and its outcome. The implication is that enhancing our grasp of how MBI change mechanisms occur enables clinicians to focus more precisely on essential EF elements while eliminating ineffective targets. Such efforts might lead to more effective treatments that offer quicker and stronger clinical enhancements (Maddock & Blair, 2021).

Only a few RCTs examined EF as a mediator of the effect of MBIs. In Noone and Hogan (2018), performance-based EF did not mediate the effect of MBI on critical thinking and associated skills in undergraduates. Relatedly, in a cross-sectional study, higher trait mindfulness predicted more positive affect and less negative affect via better self-reported EF in everyday contexts in undergraduates (Short, Mazmanian, Oinonen, & Mushquash, 2016). However, both studies recruited unselected undergraduates. Unselected samples are problematic since task-based EF and AC have been shown to correlate frequently with high levels of psychiatric symptoms (Abramovitch, Short, & Schweiger, 2021).

Improvement in AC and EF could plausibly be a proxy change mechanism of an MBI, and such changes would precede the alleviation of worry and other repetitive negative thinking (RNT) tendencies for people with generalized anxiety disorder (GAD). *Attentional control theory* (ACT; Eysenck & Derakshan, 2011) and the *cognitive model* (Hirsch & Mathews, 2012) posit that EF and associated issues, such as biased attention toward threat and unhelpful interpretations, generate excessive and uncontrollable worry (core GAD symptoms). Buttressing ACT, a meta-analysis showed negative correlations between rumination and both inhibition (Pearson's *r* = −0.23) and set-shifting (*r* = −0.19; Yang, Cao, Shields, Teng, and Liu, 2017). Supporting the cognitive model, compromised set-shifting, inhibition, WM, and inductive reasoning abilities predicted GAD diagnosis and increased symptom severity after nine years among community adults (Zainal & Newman, 2018).

Additionally, *scar theories* (McEwen & Gianaros, 2011; Ottaviani et al., 2016) posit that worry and RNT trigger persistent activation and disturbance of interconnected neuroendocrine and immune regulatory systems that may build up allostatic load over time. Allostatic load is defined as the gradual deterioration of the hypothalamic-pituitary-adrenal axis (HPA) and related systems across time (McEwen & Seeman, 1999), potentially impacting EF-implicated brain areas (Juster, McEwen, & Lupien, 2010). Concurring with scar theories, heightened excessive worry and other GAD symptoms predicted future EF declines (Zainal & Newman, 2021), and increased inflammation consistently mediated this prospective association in separate samples (Zainal & Newman, 2022a, 2022b). Collectively, since AC and EF issues are bidirectionally related to pathological worry and other RNT habits, MBIs might be efficacious for GAD by improving AC and EF as proxy change mechanisms.

On that note, typical MBIs included 8–16 weeks of mindfulness-based stress reduction (MBSR; Kabat-Zinn, 1990) and mindfulness-based cognitive therapy (MBCT; Williams, Russell, & Russell, 2008), coupled with daylong meditation retreats (Creswell, 2017). Nevertheless, research uniformly showed that most people with GAD would not seek out and attend traditional face-to-face psychotherapy (including MBIs; e.g. Olfson, Blanco, Wall, Liu, & Grant, 2019), and some of them would instead prefer to solve their mental health struggles independently (Goetter et al., 2020; Rackoff, Fitzsimmons-Craft, Taylor, Wilfley, & Newman, 2023). Scalable, evidence-based mental health apps (or ecological momentary interventions; EMIs) might somewhat solve this issue (Marciniak et al., 2020). EMIs use experience sampling methods to offer individualized assistance in real-time by recognizing a person's inner and outer contexts and emotional struggles (Henry et al., 2024). Mindfulness EMIs (MEMIs), particularly those with mood-tracking attributes, exhibited modest yet notable efficacy against self-monitoring placebos (SM) on GAD symptoms (cf. recent meta-analysis by Linardon et al., 2024). These findings suggested that a MEMI against SM might reduce GAD symptoms and RNT tendencies by enhancing AC and EF.

The current study was a secondary analysis of an RCT for GAD. In prior reports, a 14-day MEMI, compared to SM, reduced RNT and GAD severity (Zainal & Newman, 2024c, 2023b) and enhanced various empathy domains (Zainal & Newman, 2024a) to a greater degree from pre-treatment to one-month follow-up (1MFU). Two new hypotheses based on theory and evidence reviewed were tested. Hypothesis 1 predicted that a MEMI would significantly outperform SM in enhancing pre-post AC, inhibition, set-shifting, VF, and WM. Hypothesis 2 predicted that the effect of a MEMI against SM on reducing pre-1MFU GAD severity and trait RNT would be significantly mediated by improved pre-post AC, inhibition, set-shifting, VF, and WM.

## Method

### Study design

**Figure 1.**
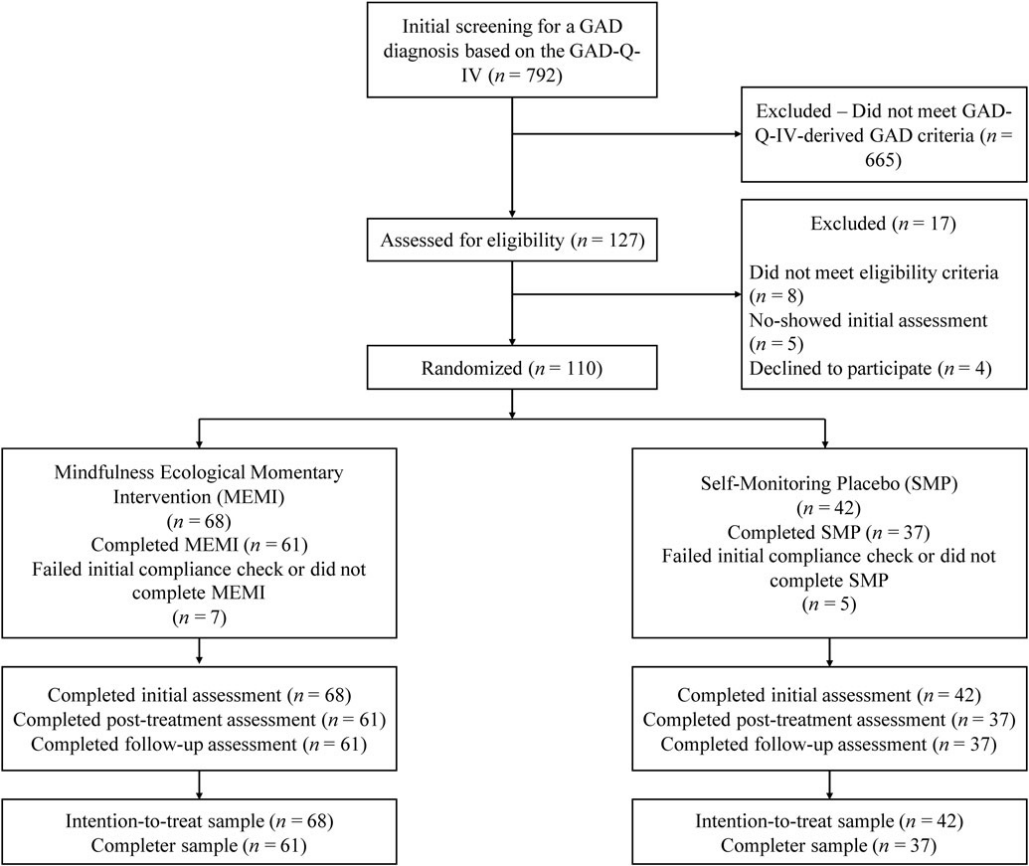
CONSORT flowchart of participant recruitment and progress. *Note:* CONSORT, Consolidated Standards of Reporting Trials; GAD, generalized anxiety disorder; GADQ-IV, GAD questionnaire- fourth edition; MEMI, mindfulness ecological momentary intervention; SMP, self-monitoring app or placebo.

The Pennsylvania State University Institutional Review Board granted ethical permission to conduct our study. Our pre-registered RCT (NCT04846777 and https://osf.io/7g4su) used a mixed design involving two treatments (MEMI and SM) and three time points (pre-randomization, post-intervention, and 1MFU). Online Supplementary Appendix A offers an extensive summary of the methodology, detailing compensation details, power analysis, and pre-randomization measures. Figure 1 presents the CONSORT (Consolidated Standards of Reporting Trials) diagram illustrating participant flow from enrollment through study completion (Montgomery et al., 2018).

### Eligibility criteria

Individuals were recruited from the subject pool and local community via initial screening of GAD criteria based on the Generalized Anxiety Disorder Questionnaire-IV (GADQ-IV; Newman et al., 2002), a minimum age of 18 years, and ownership of an iPhone or Android smartphone. They also had to be actively seeking treatment but not presently receiving any intervention (e.g. psychotropic medications) for mental health. Relatedly, they had to be meditation-naïve and report no prior experience with structured mindfulness practices. Those who consented were invited to participate in a 30-minute clinical interview using the Anxiety and Related Disorders Interview Schedule for DSM-5 (ADIS-5; Brown & Barlow, 2014) to confirm Diagnostic and Statistical Manual-Fifth Edition Text Revision (DSM-5-TR; American Psychiatric Association, 2022) GAD diagnosis. Exclusion criteria were presence of substance abuse disorders, suicidal thoughts, manic episodes, or psychotic disorders.

### Participants

We enrolled a total of 110 participants diagnosed with GAD and meeting study eligibility criteria; 42 were randomly assigned to SM and 68 to MEMI. Table 1 presents the attributes of recruited participants. There was no between-group difference in baseline diagnoses (alcohol use disorder, anorexia nervosa, binge-eating disorder, major depressive episode [current or recurrent], obsessive-compulsive disorder [OCD], panic disorder, post-traumatic stress disorder [PTSD], social anxiety disorder [SAD], substance use disorder).

**Table 1.** Sociodemographic data of study participants in the mindfulness ecological momentary intervention (MEMI) and self-monitoring app (SM) (*N* = 110)

	MEMI (*n* = 68)		SM (*n* = 42)		*p*
Continuous variables	*M*	(*SD*)	*M*	(*SD*)	
Age (in years)	20.53	(3.91)	21.24	(7.24)	.51
14-item GAD-Q-IV score	9.52	(2.10)	9.94	(1.96)	.30
Treatment expectations					
Credibility	6.00	(1.39)	5.72	(1.58)	.34
Expectancy	43.46	(17.33)	44.29	(18.13)	.31
Categorical variables	*n*	(%)	*n*	(%)	*p*
Gender orientation					.85
Declined to disclose	1	(1.47)	–	–	
Women	57	(83.82)	37	(88.10)	
Men	10	(14.71)	5	(11.90)	
Race					.99
African American	5	(7.35)	1	(2.38)	
Another race	4	(5.88)	2	(4.76)	
Asian or Asian American	11	(16.18)	4	(9.52)	
Declined to disclose	1	(1.47)	0	(0.00)	
Hispanic	3	(4.41)	5	(11.91)	
White Caucasian	44	(64.71)	27	(64.29)	
Comorbid diagnoses					
Current alcohol use disorder	7	(10.30)	1	(2.38)	.12
Current anorexia nervosa	0	(0.00)	0	(0.00)	–
Current binge-eating disorder	1	(1.47)	0	(0.00)	.39
Current major depressive episode	32	(47.10)	24	(57.10)	.30
Recurrent major depressive episode	25	(36.80)	20	(47.60)	.26
Current OCD	4	(5.88)	4	(9.52)	.48
Current panic disorder	16	(23.50)	5	(11.90)	.13
Current PTSD	9	(13.20)	4	(9.52)	.56
Current social anxiety disorder	15	(22.10)	14	(33.30)	.19
Current substance use disorder	3	(4.41)	1	(2.38)	.58

*Note*. GAD-Q-IV, generalized anxiety disorder questionnaire-fourth edition; OCD, obsessive-compulsive disorder; PTSD, post-traumatic stress disorder.

### Pre–post mediator measures

#### AC

The Attentional Control Scale (ACS; Derryberry & Reed, 2002) comprised 20 self-assessed items, combining a 9-item attentional focus measure with an 11-item attentional shifting subtest. It demonstrated strong convergent validity (agreement with related measures), good predictive validity (Judah, Grant, Mills, & Lechner, 2014), satisfactory discriminant validity (differentiation from unrelated constructs; Williams, Rau, Suchy, Thorgusen, & Smith, 2017), and high retest reliability (Abasi, Mohammadkhani, Pourshahbaz, & Dolatshahi, 2017). The alpha (internal consistency) values in the present study were .87, .90, and .90 at pre-randomization, post-intervention, and 1MFU, respectively. Higher scores denoted better AC skills.

#### Inhibition

The Delis-Kaplan Executive Function System (D-KEFS; Delis, Kaplan, & Kramer, 2001) Color-Word Inhibition Test (CWIT) Condition 3, a modification of the classic Stroop test (Stroop, 1935), measured performance-based inhibition. Participants viewed a matrix of 50 color words with three ink colors (blue, red, and green). They were directed to verbalize the ink color as quickly and accurately as possible and to abstain from naming the word. The CWIT offers an advantage in measuring inhibition by eliminating the confounding effect of triggering symptoms with emotional words (Hsu & Davison, 2017). CWIT scores were correlated with GAD symptoms (Beaudreau et al., 2017; Dorenkamp, Irrgang, & Vik, 2023). The D-KEFS CWIT had good reliability and construct validity in assessing EF (Delis, Kramer, Kaplan, & Holdnack, 2004; Homack, Lee, & Riccio, 2005). Higher scores were indicative of poorer performance.

#### Set-shifting

The paper-based Trail Making Test-B (TMT-B) assessed set-shifting, attention, and processing speed (Army Individual Test Battery, 1944). Participants were required to alternately connect numbers and letters sequentially in ascending order as quickly as possible, with the assessor recording their completion time or speed (Spreen & Strauss, 1998). The TMT-B had good retest reliability (Bracken, Mazur-Mosiewicz, & Glazek, 2019) and strong convergent validity (Osuka, Kojima, Sakurai, Watanabe, & Kim, 2020; Plotnik et al., 2017). Higher TMT-B scores indicated poorer set-shifting skills.

#### VF

VF was assessed via the D-KEFS VF subtest, comprising three progressively challenging subtests (Delis et al., 2001). This encompassed Letter Fluency (swiftly naming words beginning with a designated letter), Category Fluency (swiftly naming words within a specified domain), and Category Switching (promptly transitioning between semantic domains). The D-KEFS VF subtest evidenced good psychometric reliability and construct validity (Suchy & Brothers, 2022). Participants were allotted 1 min in each test to generate as many words as possible. Higher sum scores on these subtests indicated better VF ability.

#### WM

Letter-number sequencing (LNS) in the Wechsler-Adult Intelligence Scale Fourth Edition (WAIS-IV; Wechsler, 2008) was used because it is considered the best WM assessment (Crowe, 2000; Salthouse, 1996; Shelton, Elliott, Hill, Calamia, & Gouvier, 2009). The evaluator verbally presented a sequence of alphanumeric strings at a pace of about one string per second. Participants were instructed to listen and reiterate successively longer alphanumeric strings by verbalizing the numbers before the letters in ascending order (Reynolds, 1997). LNS captured WM, auditory processing speed, attention span, and cognitive manipulation. It has strong internal consistency, retest reliability, and construct validity (Vora, Varghese, Weisenbach, & Bhatt, 2016). The sum of correct responses from all three trials was calculated, and higher scores reflected better WM ability.

### Pre-1MFU outcome measures

#### GAD severity

GAD symptom severity was evaluated using the 16-item GAD-Q-Dimensional assessment, which closely resembled and was adapted from the GAD-Q-IV (Newman et al., 2002) but consistently used 9-point Likert scale response options (e.g. 0 = *Never* to 8 = *Almost Every Day*). The first eight items assessed individuals' levels of trait anxiety, with respondents evaluating the extent, frequency, intensity, and manageability of their worry across their lifetime. The subsequent eight questions posed similar queries regarding the preceding half-year (*α* values: .96, .97, .97).

#### Trait RNT

The 45-item Perseverative Cognitions Questionnaire (PCQ) measured enduring cognitive attributes associated with brooding, obsessive thinking, and worry (Szkodny & Newman, 2019). Participants rated items on a 6-point Likert scale (0 = *Strongly Disagree* to 5 = *Strongly Agree*). The PCQ has six factors: anticipating adverse outcomes, exploring reasons and meanings, perceiving lack of control, preparing for the future, ruminating on past events, and thoughts incongruent with one's ideal self. The overall score was determined by summing items of each subscale. It had robust retest reliability over two weeks, strong convergent and discriminant validity (Szkodny & Newman, 2019), and good cross-cultural equivalence (Zainal, Newman, & Hong, 2021; *α* values = .96, .97, .97).

### Group arms

#### MEMI

MEMI participants accessed a video featuring the principal researcher, a doctoral-level clinical psychologist. The video taught them to use evidence-supported components in accordance with the principles derived from MBSR (Kabat-Zinn, 1990). They were introduced to the concept of mindfulness and given precise guidance to immerse themselves in their immediate environment and actively participate in *meaningful activities*. This portion was designed to give them the capability of *open monitoring and acceptance*, improving their capacity to concentrate on intricate details. Following that, the video therapist guided unhurried *diaphragmatic breathing* techniques with a practical demonstration of the optimal procedure. This segment encompassed instructions on cultivating tranquility via optimized breathing routines and nurturing mindfulness qualities such as *non-reactive observation and non-judgment*, borrowing inspiration from the principles of MBCT (Segal, Williams, & Teasdale, 2002). Subsequently, the video therapist stressed *integrating mindfulness into daily activities*. The instructional material explicitly asked them to consistently review and actively participate in mindfulness via MEMI exercises (online Supplementary Appendix B).

#### Self-monitoring app (SM)

The SM video commenced with an introduction to self-awareness as the heightened recognition of one's feelings and thinking states. Subsequently, SM suggested that practicing self-observation and recording of thoughts emotional discomfort could potentially aid in nurturing more beneficial thought-feeling repertoires. It conveyed that self-observation by itself could reduce anxiety symptoms. The core foundation was derived and adapted from the rationale employed in a recent app-based intervention (LaFreniere & Newman, 2016, 2020). Therefore, it purposefully avoided any reference to the concept of mindfulness. SM avoided any directives to enhance awareness and perception of current experiences. Instead, it concentrated on monitoring distressing thoughts and feelings. Participants were not guided to focus exclusively on their ongoing tasks or be in the moment. Although SM participants were encouraged to observe distress-associated thoughts and feelings, we omitted any instructions regarding accepting cognitive-affective states as they arose. SM also did not offer guidance on techniques for regulating and optimizing breath or prolonging self-observation practices after the 14-day intervention phase ended (online Supplementary Appendix C). This placebo control approach sought to increase credibility of the SM and to prevent any potential amplification of between-group effect sizes observed with a no-treatment/waitlist control (Lutz, Offidani, Taraboanta, Lakhan, & Campellone, 2022).

### Procedures

Those meeting eligibility criteria completed a series of initial self-assessments and participated in behavioral tests capturing EF. After completing the 14-day intervention (with five prompts/day), all participants completed the same measures at post-intervention and 1MFU (six weeks from the start). Measures were counter-balanced to prevent order effects. To maintain assessor-blinding to randomized arms, assessors either physically left the room (before COVID-19) or instructed participants to mute their Zoom before accessing the designated video link (during and post-COVID-19). Participants installed the PACO mobile application, which came preloaded with MEMI or SM (https://github.com/google/paco). The assessor answered questions concerning protocols, such as upcoming appointments or technical problems regarding installing the PACO app on their mobile devices. Nonetheless, the assessor did not attend when participants were informed about their assigned treatment and its constituents. Participants were provided a MEMI or SM intervention rationale document automatically delivered via Qualtrics to uphold assessor-blinding. They received compensation in credit hours, monetary remuneration, or their mixture (online Supplementary Appendix A). On the seventh intervention day, the team conducted an assessment to verify whether participants had adhered to the instruction of completing a minimum of 56 out of 70 prompts.

### Data analyses

Random forest imputation utilizing the *missRanger R* package was employed to address missing data, which accounted for 11% of the dataset (Mayer, 2023). To evaluate MEMI against SM concerning their effects on specific EF mediators, we applied an intent-to-treat approach similar to the primary efficacy analysis (Zainal & Newman, 2023b). This entailed a two-tiered multilevel model, which examined changes from pre-treatment to 1MFU in GAD severity or trait RNT, with group as the between-person factor. We utilized a counterfactual causal approach in multilevel mediation (online Supplementary Appendix A and VanderWeele, 2016).

The analysis examined three multiplicative routes: effect of treatment group assignment on the pre–post mediator (*a* path), pre-post mediator's effect on pre-1MFU outcome (*b* path), and treatment group effect on pre-1MFU outcome (*c* path or direct effect). Simultaneously adjusting for treatment group effect, the mediation effect is called the indirect effect (VanderWeele, 2016). Temporal precedence was set such that treatment assignment preceded the pre–post EF mediator, and the pre–post EF mediator preceded the pre-1MFU endpoint (Winer et al., 2016). Given the theoretical significance of each plausible mediator and their interconnectedness, we refrained from adjusting for additional mediators (Vansteelandt & Daniel, 2017). We displayed the non-standardized regression coefficients (*β*) with 95% confidence intervals (CIs) and used bootstrapping with 1000 resampling repetitions (Cheung & Lau, 2008). We performed sensitivity assessments using non-linear generalized additive multilevel models to examine the extent to which the observed results remained consistent (Imai, Keele, & Tingley, 2010). Effect size was calculated as proportion of the indirect effect in relation to the total effect (Wen & Fan, 2015). For mediation analyses, we employed three *R* packages: *intmed* (Chan, 2020), *mgcv* (Wood, 2014), and *mediation* (Tingley, Yamamoto, Hirose, Keele, & Imai, 2014) as well as adapted published tutorials for our analyses (http://tinyurl.com/missRanger; http://tinyurl.com/codesintmed; http://tinyurl.com/codesmediation).

## Results

### Path *a*: random assignment predicting each pre–post EF mediator

**Figure 2.**
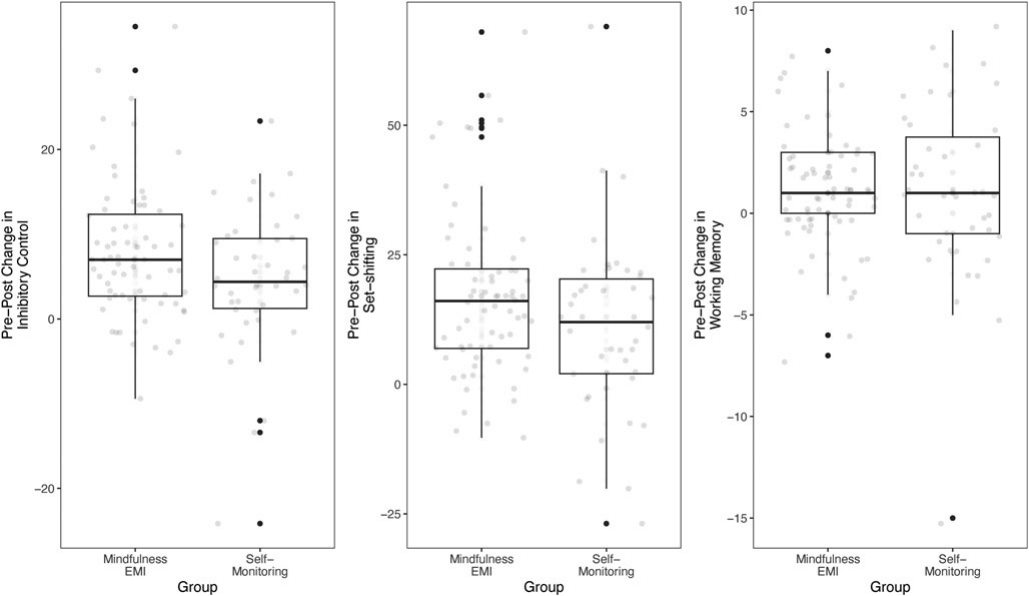
Significant comparative efficacy of mindfulness EMI against SM on pre-post EF domains. *Note:* EF, executive functioning; EMI, ecological momentary intervention; SM, self-monitoring placebo.

MEMI v. SM had significantly stronger effects on enhancing pre-post inhibition (*β* = −2.075, *p* = .002), WM (*β* = 0.512, *p* = .045), and set-shifting (*β* = −2.916, *p* = .010), but not VF (*β* = −0.991, *p* = .547) and AC (*β* = 0.866, *p* = .337; Figure 2). MEMI yielded significant within-group pre-post improvements in inhibition (*β* = −3.958, *p* < .001), VF (*β* = 4.331, *p* = .004), WM (*β* = 0.551, *p* = .017), and set-shifting (*β* = −8.566, *p* < .001), but not AC (*β* = 1.201, *p* = .151). By comparison, SM produced significant within-group pre-post enhancements in inhibition (*β* = −2.276, *p* = .002) and set-shifting (*β* = −8.566, *p* < .001) but not VF (*β* = 4.179, *p* = .065), WM (*β* = 0.571, *p* = .103), or AC (*β* = 1.201, *p* = .151; Table 2).

**Table 2.** Simple slope analyses of counterfactual mediation analysis of EF domains mediating the effect of MEMI against SM on pre-1MFU GAD severity

	Predicting pre–post mediator (*a* path)			Predicting pre-1MFU GAD severity (*b* path)		
	*β*	[LCI, UCI]	*p*	*β*	[LCI, UCI]	*p*
A. Inhibitory control						
Intercept (MEMI)	43.351***	[41.587, 45.114]	.000	58.228***	[43.385, 73.071]	.000
Time (MEMI)	−3.958***	[−5.324, −2.592]	.000	−5.072**	[−8.575, −1.569]	.005
Intercept (SM)	44.005***	[42.159, 45.851]	.000	81.062***	[57.813, 104.311]	.000
Time (SM)	−2.276**	[−3.705, −0.846]	.002	−1.076	[−5.412, 3.259]	.627
B. Verbal fluency						
Intercept (MEMI)	113.194***	[109.383, 117.004]	.000	68.234***	[50.296, 86.173]	.000
Time (MEMI)	4.331**	[1.380, 7.282]	.004	−6.996***	[−10.357, −3.634]	.000
Intercept (SM)	115.099***	[109.421, 120.777]	.000	78.524***	[58.554, 98.494]	.000
Time (SM)	4.179	[−0.220, 8.577]	.065	−1.083	[−5.315, 3.150]	.617
C. Working memory (WM)						
Intercept (MEMI)	19.934***	[19.352, 20.515]	.000	72.373***	[51.792, 92.954]	.000
Time (MEMI)	0.551*	[0.101, 1.002]	.017	−6.798***	[−10.145, −3.452]	.000
Intercept (SM)	19.048***	[18.168, 19.927]	.000	78.825***	[57.584, 100.065]	.000
Time (SM)	0.571	[−0.11, 1.253]	.103	−1.064	[−5.284, 3.156]	.622
D. Set-shifting						
Intercept (MEMI)	55.033***	[52.056, 58.009]	.000	72.787***	[61.124, 84.449]	.000
Time (MEMI)	−8.566***	[−10.872, −6.261]	.000	−6.193***	[−9.899, −2.487]	.001
Intercept (SM)	54.845***	[51.679, 58.011]	.000	68.333***	[51.175, 85.491]	.000
Time (SM)	−5.537***	[−7.989, −3.085]	.000	0.161	[−4.296, 4.618]	.944
E. Attentional control						
Intercept (MEMI)	53.591***	[51.482, 55.701]	.000	100.537***	[85.413, 115.662]	.000
Time (MEMI)	1.201	[−0.432, 2.835]	.151	−6.148***	[−9.379, −2.916]	.000
Intercept (SM)	51.768***	[48.712, 54.824]	.000	102.896***	[86.485, 119.306]	.000
Time (SM)	1.430	[−0.937, 3.796]	.239	−0.396	[−4.461, 3.668]	.849

*Note.* * *p* < 0.05; ** *p* < 0.01; *** *p* < 0.001.

EF, executive functioning; MEMI, ecological momentary intervention; SM, self-monitoring control; 1MFU, one-month follow-up; *β*, unstandardized regression estimate; GAD, generalized anxiety disorder; LCI, lower limit of the 95% confidence interval (CI); UCI, upper limit of the 95% CI. All *b* path (mediator-outcome associations) models adjusted for the specific pre-post mediator.

### Path *b*: pre–post EF predicting pre-1MFU change in GAD severity

Treatment group assignment did not significantly moderate the association of pre-post change in inhibition (*β* = 0.565, *p* = .057), VF (*β* = 0.011, *p* = .924), WM (*β* = −0.126, *p* = .867), set-shifting (*β* < 0.001, *p* = .999), and AC (*β* = −0.063, *p* = .760) predicting pre-1MFU change in GAD severity. Within the MEMI arm, pre-post improvements in inhibition (*β* = −5.072, *p* = .005), VF (*β* = −6.996, *p* < .001), WM (*β* = −6.798, *p* < .001), set-shifting (*β* = −6.193, *p* = .001), and AC (*β* = −6.148, *p* < .001) significantly predicted pre-1MFU declines in GAD severity (Table 2). However, within the SM arm, pre-post change in inhibition (*β* = −1.076, *p* = .627), VF (*β* = −1.083, *p* = .617), WM (*β* = −1.064, *p* = .622), set-shifting (*β* = 0.161, *p* = .944), and AC (*β* = −0.396, *p* = .849) did not significantly predict pre-1MFU change in GAD severity.

### Path *b*: pre–post EF predicting pre-1MFU change in trait RNT

Treatment group assignment significantly moderated the association of pre-post inhibition (*β* = 0.036, *p* = .003) predicting pre-1MFU change in trait RNT. However, treatment group did not moderate the association of pre-post VF (*β* < 0.001, *p* = .832), WM (*β* = −0.042, *p* = .172), set-shifting (*β* = 0.007, *p* = .316), and AC (*β* = 0.003, *p* = .708) predicting pre-1MFU change in trait RNT. Within the MEMI arm, pre-post improvements in inhibition (*β* = −0.236, *p* = .002), VF (*β* = −0.309, *p* < .001), WM (*β* = −0.300, *p* < .001), set-shifting (*β* = −0.278, *p* < .001), and AC (*β* = −0.283, *p* < .001) significantly predicted pre-1MFU declines in trait RNT (Table 3). However, within the SM arm, pre-post change in inhibition (*β* = −0.100, *p* = .261), VF (*β* = −0.055, *p* = .521), WM (*β* = −0.076, *p* = .378), set-shifting (*β* = −0.059, *p* = .524), and AC (*β* = −0.028, *p* = .715) did not significantly predict pre-1MFU change in trait RNT.

**Table 3.** Simple slope analyses of counterfactual mediation analysis of EF domains mediating the effect of MEMI against SM on pre-1MFU trait repetitive thinking

	Predicting pre-post mediator (*a* path)			Predicting pre-1MFU trait repetitive thinking (*b* path)		
	*β*	[LCI, UCI]	*p*	*β*	[LCI, UCI]	*p*
A. Inhibitory control						
Intercept (MEMI)	43.351***	[41.587, 45.114]	.000	2.111***	[1.501, 2.721]	.000
Time (MEMI)	−3.958***	[−5.324, −2.592]	.000	−0.236**	[−0.380, −0.092]	.002
Intercept (SM)	44.005***	[42.159, 45.851]	.000	3.584***	[2.657, 4.51]	.000
Time (SM)	−2.276**	[−3.705, −0.846]	.002	−0.100	[−0.272, 0.073]	.261
B. Verbal fluency						
Intercept (MEMI)	113.194***	[109.383, 117.004]	.000	3.220***	[2.476, 3.963]	.000
Time (MEMI)	4.331**	[1.380, 7.282]	.004	−0.309***	[−0.448, −0.170]	.000
Intercept (SM)	115.099***	[109.421, 120.777]	.000	3.668***	[2.875, 4.461]	.000
Time (SM)	4.179	[−0.220, 8.577]	.065	−0.055	[−0.223, 0.113]	.521
C. Working memory (WM)						
Intercept (MEMI)	19.934***	[19.352, 20.515]	.000	3.613***	[2.765, 4.461]	.000
Time (MEMI)	0.551*	[0.101, 1.002]	.017	−0.300***	[−0.438, −0.163]	.000
Intercept (SM)	19.048***	[18.168, 19.927]	.000	3.081***	[2.231, 3.931]	.000
Time (SM)	0.571	[−0.11, 1.253]	.103	−0.076	[−0.245, 0.093]	.378
D. Set-shifting						
Intercept (MEMI)	55.033***	[52.056, 58.009]	.000	2.752***	[2.270, 3.234]	.000
Time (MEMI)	−8.566***	[−10.872, −6.261]	.000	−0.278***	[−0.431, −0.125]	.000
Intercept (SM)	54.845***	[51.679, 58.011]	.000	2.948***	[2.256, 3.640]	.000
Time (SM)	−5.537***	[−7.989, −3.085]	.000	−0.059	[−0.238, 0.121]	.524
E. Attentional control						
Intercept (MEMI)	53.591***	[51.482, 55.701]	.000	4.517***	[3.912, 5.122]	.000
Time (MEMI)	1.201	[−0.432, 2.835]	.151	−0.283***	[−0.413, −0.154]	.000
Intercept (SM)	51.768***	[48.712, 54.824]	.000	4.838***	[4.241, 5.436]	.000
Time (SM)	1.430	[−0.937, 3.796]	.239	−0.028	[−0.176, 0.120]	.715

*Note.* * *p* < 0.05; ** *p* < 0.01; *** *p* < 0.001.

EF, executive functioning; MEMI, ecological momentary intervention; SM, self-monitoring control; 1MFU, one-month follow-up; *β*, unstandardized regression estimate; LCI, lower limit of the 95% confidence interval (CI); UCI, upper limit of the 95% CI.

### Indirect effect: treatment effect on pre-1MFU change in GAD severity via EF

The effect of MEMI compared to SM on reduced pre-1MFU GAD severity was significantly mediated by enhanced pre-post inhibition performance (*β* = −0.605, *p* = .030; proportion mediated = 7.1%). However, pre-post AC (*β* = −0.409, *p* = .340), set-shifting (*β* = −0.302, *p* = .236), VF (*β* = −0.046, *p* = .746), and WM (*β* = 0.045, *p* = .808) were non-significant mediators of the effect of MEMI against SM on decreased pre-1MFU GAD severity. Effect sizes ranged from 0.2% to 4.7% for non-significant mediation paths.

### Indirect effect: treatment effect on pre-1MFU change in trait RNT via EF

The effect of MEMI compared to SM on decreased pre-1MFU trait RNT was significantly mediated by enhanced pre-post inhibition performance (*β* = −0.024, *p* = .040; proportion mediated = 7.4%). However, pre-post AC (*β* = 0.005, *p* = .900), set-shifting (*β* = −0.012, *p* = .222), VF (*β* = 0.003, *p* = .570), and WM (*β* = −0.008, *p* = .308) were non-significant mediators of the effect of MEMI against SM on reduced pre-1MFU trait RNT. Effect sizes ranged from 0% to 3.6% for non-significant mediation paths.

## Discussion

Partially supporting Hypothesis 1, MEMI generated greater pre-post enhancements in inhibition, WM, and set-shifting but not VF and AC. Although there was no significant difference between MEMI and SM on increased VF, simple slope analysis revealed pre-post VF improvements in MEMI but not SM. Hypothesis 2 also received partial support, as pre-post improvement in inhibition uniquely mediated the effect of MEMI compared to SM on pre-follow-up reductions in GAD symptom severity and trait RNT. The combination of therapy elements in MEMI, such as acceptance, diaphragmatic breathing retraining, engagement with present activity, non-reactivity, and open monitoring, likely contributed to any observed differential efficacy over SM rather than any single component alone.

Other potential theoretical propositions are considered to inform optimization efforts of MBIs for GAD. Pre-post enhancements in inhibition accounted for the effect of MEMI against SM on pre-follow-up GAD severity and RNT. Further, pre-post enhancements in inhibition mediated pre-follow-up decline in RNT more strongly in MEMI than SM. Since people with GAD experience their worries as uncontrollable (Hallion & Ruscio, 2013) and have negative beliefs about worry (LaFreniere & Newman, 2019), it may be necessary to improve inhibition skills to put a brake on worry and other perseverative cognitions. Relatedly, people with GAD worry in autopilot ways that heighten and prolong distress to avoid negative emotional contrasts, i.e. sharp rises from positive or neutral to negative affect states (cf. contrast avoidance theory; Newman & Llera, 2011; Newman, Llera, Erickson, Przeworski, & Castonguay, 2013). Thus, MEMI might have fostered tolerance of intense surges in distress, relinquishing the usual disinhibited, reflexive urges to avoid or resist negative emotional contrasts and, instead, allowing these experiences to fully register in one's awareness by enhancing inhibition. Future studies should investigate this idea by examining the link between inhibition and contrast avoidance in GAD.

Additional issues in GAD could explain the salience of inhibition as a potential change mechanism (cf. attentional control theory; Eysenck, Derakshan, Santos, & Calvo, 2007). People with GAD (*v.* controls) displayed poorer decision-making on inhibition-based reinforcement learning tasks (White et al., 2017). Relatedly, higher clinician-assessed GAD severity was correlated with slower and less precise performance in the Stroop task (Hallion, Tolin, Assaf, Goethe, & Diefenbach, 2017). Inhibitory dyscontrol has been observed to be a correlate (Majeed et al., 2023), predictor (Zainal & Newman, 2018), and longitudinal outcome of increase in pathological worry (Zainal & Newman, 2023a, 2021). These problems might translate to ample opportunities for MEMI to remedy inhibition deficits in GAD, thereby alleviating future worry and other RNT. Further, pathological worry preceded and increased future inhibition deficits within individuals across time (cf. *scar theories*; Zainal & Newman, 2021). Thus, MEMI likely improved inhibition by teaching and reinforcing the skill of resisting the habit of worrying, ruminating, or obsessing (Gallant, 2016). Enhancing inhibition by focusing on the here-and-now instead of the past/future with perseverative cognitions may have led to pre-follow-up decreases in worry and other RNT propensities.

Why did MEMI yield stronger pre–post improvements in set-shifting and WM relative to SM? Perhaps MEMI liberated cognitive resources that were otherwise consumed by suppressing worry-related, task-irrelevant thoughts, leading to an overall enhancement in cognitive efficiency to deploy better set-shifting and updating WM skills (Course-Choi, Saville, & Derakshan, 2017; Jankowski & Holas, 2020). The potential for MEMI to induce better meta-cognitive skills, such as non-identification with feelings and thoughts (McEvoy, Graville, Hayes, Kane, & Foster, 2017), could also explain these results. Further, these outcomes might make sense given how patients with (*v.* without) GAD continually exhibited worse WM task performance under threat conditions (Vytal, Arkin, Overstreet, Lieberman, & Grillon, 2016). When exposed to emotion-inducing distractions, they displayed reduced activity and white matter volume in WM-linked dorsolateral prefrontal cortex areas (Moon & Jeong, 2017) and struggled with cognitively retaining materials germane to present objectives (Moon, Sundaram, Choi, & Jeong, 2016; Yoon, LeMoult, Hamedani, & McCabe, 2018). Together, MEMI might reverse worry-triggered set-shifting and WM deficits by freeing cognitive processing assets (cf. resource allocation theory; Levens, Muhtadie, & Gotlib, 2009) and instructing focus on the here-and-now and task-switching flexibility.

Nevertheless, set-shifting and WM did not mediate treatment effects. MBIs were also found to be more efficacious for accuracy (v. latency), set-shifting, and WM scores (cf. meta-analysis; Zainal & Newman, 2024b). It is possible that instead of the TMT-B, using other set-shifting measures based on accuracy rather than latency might have increased the chances of detecting a mediation effect. Likewise, WM measures apart from the WAIS-IV LNS might have been more sensitive for mediation purposes, such as the automated operation span task (Dubert, Schumacher, Locker, Gutierrez, & Barnes, 2016; Unsworth, Heitz, Schrock, & Engle, 2005). Alternatively, the lack of mediation effects with set-shifting and WM might be due to the present RCT being underpowered to detect small effect sizes for these domains (cf. method paper by Qin, 2024).

Although no between-group effects emerged, within-group analyses revealed notable pre-post improvements in VF in MEMI but not SM. These findings might be explained by evidence that brief MBIs could enhance verbal learning and memory via refinements in the ability to register information (Lueke & Lueke, 2019). Relatedly, since VF is associated with aptitude to efficiently recall words linked to emotions (Hegefeld, Satpute, Ochsner, Davidow, & Nook, 2023), MEMI might have strengthened VF of emotion and non-emotion words more than SM (Edwards, Shivaji, & Wupperman, 2018). This interpretation could be understood in the context of struggles to recognize and describe emotions in GAD (Paniccia et al., 2020). To confirm these interpretations, experimental work is required to test these conjectures.

Unexpectedly, neither between- nor within-group effects on self-reported AC occurred. Although a prior cross-sectional study showed that higher AC mediated the inverse anxiety-mindfulness correlation (MacDonald & Olsen, 2020), such findings did not extend to our longitudinal RCT of MEMI for GAD. These outcomes might be accounted for by weak correlations between self-reported and performance-based AC measures (Snyder, Friedman, & Hankin, 2021). In addition, based on recent theoretical formulations (Prakash, 2021) and evidence of the efficacy of 8-week MBSR on AC (Chin et al., 2021; Lee et al., 2021), lengthier and more rigorous forms of MEMI might be needed to improve AC for GAD. Alternatively, other measures, such as task-unrelated mind-wandering probes (Mrazek, Franklin, Phillips, Baird, & Schooler, 2013), might better capture the effect of brief MEMI on AC. Relatedly, based on a meta-analysis data of robust inverse relations between AC/EF and RNT (Mennies, Stewart, & Olino, 2021), another AC measure, such as the self- or parent-reported Behavior Rating Inventory of EF (Gioia, Isquith, Guy, & Kenworthy, 2000; Guy, Isquith, & Gioia, 2004), might have mediated treatment effects.

Interpreting results in light of the broader literature on the relations between RNT and AC/EF constructs is also essential. We tested how specific EF domains mediated the efficacy of MEMI on reductions in GAD severity and RNT. Our findings regarding treatment predictors or mediators might differ if other domain-specific RNT outcomes were examined, such as anger and depressive rumination (du Pont, Rhee, Corley, Hewitt, & Friedman, 2019) or job-related rumination (Cropley & Collis, 2020), which exhibited modest yet meaningful negative correlations with a global EF. Heterogeneity also exists in the literature, such that global EF was often (Abramovitch et al., 2021), but not always (du Pont et al., 2019), linked to the internalizing symptom constructs that subsume worry.

Several limitations deserve consideration. First, future studies should examine additional factors that might explain outcomes and maximize the potential to identify differential mediator effects in the context of GAD, such as self-reported WM (Adamis & Olatunji, 2024) and performance-based composites of various domain-specific EF tasks (e.g. anti-saccade and go-no-go tasks; Gustavson et al., 2020). The proportion mediated estimate for inhibition was 7%, which might be considered a meaningful yet small effect size (Preacher & Kelley, 2011). This magnitude prompts the question of alternative EF-related pathways through which MEMI v. SM affects the outcome (VanderWeele, 2013). Despite its sensitivity in correlating with worry symptoms in other samples with GAD (Beaudreau et al., 2017; Dorenkamp et al., 2023), the neutral CWIT measure of inhibition might not sufficiently capture inhibition skills needed to curtail experiential avoidance of negative emotions or thoughts inherent in our GAD sample. Future studies should thus assess the mediation potential of AC/EF using ambulatory assessments (Hernandez et al., 2023) or AC/EF tasks that capture emotional states (Kalanthroff, 2024). The lack of tasks capturing emotional states might explain some of our null or small effect size findings. For instance, if the stimuli in the EF/AC tasks had been affect-based (e.g. emotional Stroop task; Smolker et al., 2022) or if clients with GAD had performed the tasks under induced anxiety or other emotional states (Azab, 2022) findings might have notably varied. Results might also have differed had other EF indices, such as cognitive flexibility (Baussay et al., 2024) and self-regulation (Short et al., 2016), been in the equation for all mediational analyses. Second, the 14-day intervention duration might have been inadequate to identify or generate mediation effects of all examined EF mediators, given null treatment effects on AC and VF. Eight-week MBI RCTs suggest that maximizing the detection of between-group differences in AC and VF could require more time and practice to improve present-mindedness and express emotions and thoughts more clearly (Chin et al., 2021). Third, future research should explore whether ongoing mindfulness practices could have yielded any distinct mediation effects during follow-up without repeated guidance through MEMI. Fourth, the inferences drawn from our study may not apply to a broader population beyond White females, emphasizing the need for future digital mental health EMI RCTs to include more culturally diverse participants.

Despite these limitations, several strengths were noteworthy. Our study adhered to rigorous CONSORT guidelines (Calvert, Brundage, Jacobsen, Schünemann, & Efficace, 2013; Montgomery et al., 2018), thus leveraging the methodological strengths of RCTs to eliminate bias and confounding sources. A placebo control and assessor-blinding to random assignment were also included, further reducing the potential for confounding and selection biases. Also, because we controlled for focus on and monitoring of thoughts and emotions, which is a powerful treatment in and of itself, we can more confidently attribute differential treatment effects to unique components of mindfulness. The engagement rates were also high, with a dropout rate (11%) far lower than the 24–50% dropout rates observed in app RCTs (Linardon, 2023; Linardon & Fuller-Tyszkiewicz, 2020). Further, the current study also enrolled a clinician-diagnosed sample with GAD, ensured adequate power, and incorporated a follow-up assessment.

In conclusion, MEMI was more efficacious than SM in enhancing pre-post inhibition, WM, and set-shifting, though it did not show superiority in AC and VF. Despite the lack of between-group differential efficacy, within-group analyses showed MEMI improved VF but not SM. Only inhibition mediated the effect of treatment on reductions in GAD severity and RNT. If replicated, the present study has possible practical applications in clinical contexts. Brief MEMIs for GAD might be optimized by prioritizing the targeting of inhibition rather than other EF domains. Several approaches could be tried to attain this goal. First, adding dialectical behavioral therapy components to MEMI by inviting clients to practice acceptance of life stressors *and* commit to inhibiting the urge to worry or ruminate might optimize brief MEMIs for GAD (Afshari et al., 2022; Vijayapriya & Tamarana, 2023). Second and related, GAD should change the tendency to worry in order to create and maintain negative moods to avoid sharp rises in negative emotions (cf. contrast avoidance theory; Newman & Llera, 2011; Newman et al., 2013). Plausibly, instructing clients with GAD to let go of worrying and allow experiences of emotional fluctuations, including negative emotional contrasts, via a higher-intensity version of MEMI might have a positive effect of improving inhibition. Third, as sustained worry induces wear-and-tear of physiological systems in ways that adversely affect EF over time (Zainal & Newman, 2022a, 2022b), MEMI should be merged with EF-enhancing physical exercise (cf. a meta-analysis; Moreau & Chou, 2019) among people with GAD. Fourth, future research should address the perennial inquiry of which subgroup with GAD would benefit most from inhibition-boosting exercises in conjunction with MEMI.

The current study received funding from the National Institute of Mental Health (NIMH) (R01 MH115128), the Pennsylvania State University RGSO Dissertation award, Penn State Susan Welch/Nagle Family Graduate Fellowship, the National University of Singapore (NUS) Development Grant, and the Association for Behavioral and Cognitive Therapies (ABCT) Leonard Krasner Student Dissertation Award.

## Supporting information

Zainal and Newman supplementary material Zainal and Newman supplementary material

## Supplementary material

The supplementary material for this article can be found at https://doi.org/10.1017/S0033291724001958.
