# Supplementary material for: Treatment as a moderator and executive function as a mediator of the effect of a mindfulness ecological momentary intervention for generalized anxiety disorder
Source: Psychol Med. 2024 Oct 15;54(13):3715–28. doi: 10.1017/S0033291724001958 (PMC11536110; doi:10.1017/S0033291724001958)
Supplement: Zainal and Newman supplementary material [file S0033291724001958sup001.docx]

**APPENDICES**

**Appendix A**

**Study Methodology Details**

**Power analysis**

According to a Monte Carlo power analysis (Arend & Schafer, 2019; Magnusson, 2018), our study possessed full statistical power (100%) to identify a notable Group × Time interaction effect, even with a modest effect size as small as Cohen's *d* of 0.2.

**Pre-Randomization Clinical Interview and Screening Measure**

**Psychiatric diagnoses.** Forty-five (40%) video recordings were subject to a second assessment by a different evaluator, unaware of the initial results. Agreement between the evaluators was outstanding for diagnosing GAD (Cohen's κ = 1.00) and ranged from satisfactory to good for other coexisting diagnoses and exclusions (average κs = 0.75–0.98). The Anxiety and Related Disorders Interview Schedule consistent with the Diagnostic and Statistical Manual-Fifth Edition (ADIS-5; Brown & Barlow, 2014) exhibited strong agreement with the Structured Clinical Interview for the Diagnostic and Statistical Manual (SCID; Spitzer et al., 1994) for conditions such as GAD, major depressive disorder, and other disorders (Shankman et al., 2018), with excellent inter-evaluator reliability (κ = .88 to 1.00) (Wade et al., 2022) and robust two-week retest consistency (Rutter & Brown, 2015). Additionally, the ADIS-5 displayed favorable levels of both convergent and discriminant validity (Gordon & Heimberg, 2011).

**GAD.** The 14-item GAD-Q-IV (Newman et al., 2002) DSM-5 GAD algorithm demonstrated strong performance in terms of sensitivity (0.78–0.81) and specificity (0.86–0.97) when compared to clinical interviews (Moore et al., 2014; Newman et al., 2002). Its validity was confirmed through significant associations with traits such as worry and anxiety while displaying weaker connections with unrelated constructs, such as depression (Newman et al., 2002). Additionally, it exhibited good retest reliability over a two-week period (Newman et al., 2002) and maintained strong internal consistency. In our current study, Cronbach's α values were .80, .89, and .91 for the GAD-Q-IV (total possible score ranged from 0–14) at pre-randomization, post-intervention, and one-month follow-up (1MFU).

**Pre-, Post-Treatment, and 1-Month-Follow-Up Self-Reports**

**GAD Severity.** The reliability and validity of the GAD Severity scale were established using a dataset of 883 participants from the psychology subject pool, characterized by a wide range of score variations. It demonstrated strong convergent validity, with substantial associations observed with the GAD-Q-IV (*r* = .87) and weak connections with unrelated traits such as spider phobia (*r* = .11) and rumination (*r* = .44). Furthermore, a confirmatory factor analysis revealed exceptional model fit statistics (χ^2^(*df* = 104) = 96.85, *p* = .678, Confirmatory Fit Index = 1.000, Root Mean Square Error of Approximation = .000, 95% CI [.000, 014], Standardized Root Mean Square Residual = .029, and Tucker-Lewis Index = 1.000).

**Repetitive thinking.** The 45-item Perseverative Cognitions Questionnaire (PCQ; Szkodny & Newman, 2019) consisted of six dimensions: *dwelling on the past* (14 items; e.g., "After I do something I cannot stop wondering if I made a mistake."); *expecting the worst* (4 items; e.g., "I believe good things are not likely to happen to me."); *lack of controllability* (5 items; e.g., "I am consumed by certain thoughts."); *thoughts discrepant with ideal self* (11 items; e.g., "I am a terrible person for having weird or gross thoughts."); *preparing for the future* (7 items; e.g., "I repeatedly think about a task to avoid any problems that may arise."); *searching for causes and meanings* (4 items; e.g., "I often think about my moods to figure out why I feel the way I feel.").

**EMI Self-Reports Across 14 Days from Pre-Post Intervention**

Current symptoms and mindfulness were evaluated through ecological momentary assessments both prior to and after participants received the mindfulness ecological momentary intervention (EMI) or self-monitoring app (SM) instructions at each signal.

**Treatment Credibility and Expectancy Questionnaire (CEQ; Devilly & Borkovec, 2000).** The 6-item CEQ gauged participants' perceptions of the treatment's credibility, ranging from 1 (*not logically sound*) to 9 (*highly logical*), and its potential to significantly alleviate symptoms, with a scale from 0% to 100%. The CEQ exhibited consistent reliability and strong internal consistency, with alpha coefficients of 0.86 and 0.88 for credibility and expectancy measures, respectively (Devilly & Borkovec, 2000).

**Remuneration structure**

Moreover, to meet their course obligations, participants were compensated with a combination of up to $30, 6 psychology subject pool credits, or a blend of both forms of reimbursement, adjusted in proportion to their level of engagement and completion of measures. Subject pool participants who exclusively received credits were awarded 3.5 subject pool credit hours upon finishing the initial phase of the study, which concluded before the eighth day of intervention usage. Participants who satisfactorily fulfilled study requirements up to the seventh-day compliance evaluation were invited to proceed with the study's second segment and were granted an extra 2.5 subject pool credit hours upon completion.

**Causal mediation analyses**

Traditional mediation models (e.g., Preacher et al., 2007) presume that unmeasured covariates do not influence the links between mediator and outcome (i.e., "sequential ignorability"; Cuartas & McCoy, 2021). The causal mediation method computed the difference in outcomes between a group of treatment-exposed individuals and an imaginary group of non-exposed individuals, who served as a reference for what might have occurred to the exposed individuals had they not been exposed. Since we defined the mediator as changes in plausible pre-post EF targets before the pre-1MFU outcome, participants were not assigned randomly to different mediator levels (Imai et al., 2010). Causal mediation analysis departs from the requirement for sequential ignorability by establishing a connection between causal and mediation parameters (VanderWeele, 2016).

References

Arend, M. G., & Schafer, T. (2019). Statistical power in two-level models: A tutorial based on Monte Carlo simulation. *Psychological Methods*, *24*(1), 1-19. <https://doi.org/10.1037/met0000195>

Brown, T. A., & Barlow, D. H. (2014). *Anxiety and related disorders interview schedule for DSM-5 (ADIS-5L): Client interview schedule*. Oxford University Press.

Cuartas, J., & McCoy, D. C. (2021). Causal mediation in developmental science: A primer. *International Journal of Behavioral Development*, *45*(3), 269-274. <https://doi.org/10.1177/0165025420981640>

Devilly, G. J., & Borkovec, T. D. (2000). Psychometric properties of the credibility/expectancy questionnaire. *Journal of Behavior Therapy and Experimental Psychiatry*, *31*(2), 73-86. <https://doi.org/10.1016/S0005-7916(00)00012-4>

Gordon, D., & Heimberg, R. G. (2011). Reliability and validity of DSM-IV generalized anxiety disorder features. *Journal of Anxiety Disorders*, *25*(6), 813-821. <https://doi.org/10.1016/j.janxdis2011.04.001>

Imai, K., Keele, L., & Tingley, D. (2010). A general approach to causal mediation analysis. *Psychological Methods*, *15*(4), 309-334. <https://doi.org/10.1037/a0020761>

Lutz, J., Offidani, E., Taraboanta, L., Lakhan, S. E., & Campellone, T. R. (2022). Appropriate controls for digital therapeutic clinical trials: A narrative review of control conditions in clinical trials of digital therapeutics (DTx) deploying psychosocial, cognitive, or behavioral content. *Frontiers in Digital Health*, *4*, 823977. <https://doi.org/10.3389/fdgth.2022.823977>

Magnusson, K. (2018). powerlmm: Power analysis for longitudinal multilevel models. R package version 0.4.0. <https://CRAN.R-project.org/package=powerlmm>.

Moore, M. T., Anderson, N. L., Barnes, J. M., Haigh, E. A. P., & Fresco, D. M. (2014). Using the GAD-Q-IV to identify generalized anxiety disorder in psychiatric treatment seeking and primary care medical samples. *Journal of Anxiety Disorders*, *28*(1), 25-30. <https://doi.org/10.1016/j.janxdis.2013.10.009>

Newman, M. G., Zuellig, A. R., Kachin, K. E., Constantino, M. J., Przeworski, A., Erickson, T., & Cashman-McGrath, L. (2002). Preliminary reliability and validity of the Generalized Anxiety Disorder Questionnaire-IV: A revised self-report diagnostic measure of generalized anxiety disorder. *Behavior Therapy*, *33*(2), 215-233. <https://doi.org/10.1016/S0005-7894(02)80026-0>

Preacher, K. J., Rucker, D. D., & Hayes, A. F. (2007). Addressing moderated mediation hypotheses: Theory, methods, and prescriptions. *Multivariate Behavioral Research*, *42*(1), 185-227. <https://doi.org/10.1080/00273170701341316>

Rutter, L. A., & Brown, T. A. (2015). Reliability and validity of the dimensional features of generalized anxiety disorder. *Journal of Anxiety Disorders*, *29*, 1-6. <https://doi.org/10.1016/j.janxdis.2014.10.003>

Shankman, S. A., Funkhouser, C. J., Klein, D. N., Davila, J., Lerner, D., & Hee, D. (2018). Reliability and validity of severity dimensions of psychopathology assessed using the Structured Clinical Interview for DSM-5 (SCID). *International Journal of Methods in Psychiatric Research*, *27*(1). <https://doi.org/10.1002/mpr.1590>

Spitzer, R. L., Williams, J. B. W., Kroenke, K., Linzer, M., deGruy, F. V., 3rd, Hahn, S. R., . . . Johnson, J. G. (1994). Utility of a new procedure for diagnosing mental disorders in primary care: The PRIME-MD 1000 study. *Journal of the American Medical Association*, *272*(22), 1749-1756. <https://doi.org/10.1001/jama.1994.03520220043029>

Szkodny, L. E., & Newman, M. G. (2019). Delineating characteristics of maladaptive repetitive thought: Development and preliminary validation of the Perseverative Cognitions Questionnaire. *Assessment*, *26*(6), 1084-1104. <https://doi.org/10.1177/1073191117698753>

VanderWeele, T. J. (2016). Explanation in causal inference: developments in mediation and interaction. *International Journal of Epidemiology*, *45*(6), 1904-1908. <https://doi.org/10.1093/ije/dyw277>

Wade, E. C., Cohen, R. T., Loftus, P., & Ruscio, A. M. (2022). A novel measure of real-time perseverative thought. *Clinical Psychological Science*, *10*(3), 534-552. <https://doi.org/10.1177/21677026211038017>

Zainal, N. H., & Newman, M. G. (2023). Mindfulness enhances cognitive functioning: a meta-analysis of 111 randomized controlled trials. *Health Psychology Review*, 1-27. <https://doi.org/10.1080/17437199.2023.2248222>

**Appendix B**

**Details of Intervention Arm**

The mindfulness EMI encouraged individuals to engage in daily mindfulness practices at five distinct daily intervals: around 9 a.m., noon, 3 p.m., 6 p.m., and 9 p.m., over two weeks, totaling 14 days (Appendix B). Individuals were given conventional instructions within every MEMI prompt: "Pay attention to your breathing. Breathe in a slow, steady, and rhythmic manner. Stay focused on the sensations of the air coming into your lungs and then letting it out. As you are breathing, observe your experience as it is. Let go of judgments that do not serve you. Focus on the here and now. Attend to the small moments right now (e.g., reading a chapter, having a cool glass of water), as that is where enjoyment, peace, and serenity in life happen." Prior to and following each prompt, participants assessed their current mindfulness ("To what extent are you experiencing the present moment fully?"), anxiety, and depression state levels ("To what degree do you feel keyed up or on edge [depressed] right now?") on a 9-level scale, ranging from 1 (*Not At All*) to 9 (*Extremely*). Every mindfulness EMI notification concluded with an uplifting message to promote the enduring incorporation of these abilities: "Remember that the cultivation of mindfulness is lifelong. The goal of therapy is to be your own therapist. Practice mindfulness between the prompts and after you have completed this study."

# Screenshots of Intervention Arm

## Screenshots for mindfulness ecological momentary intervention (MEMI) arm


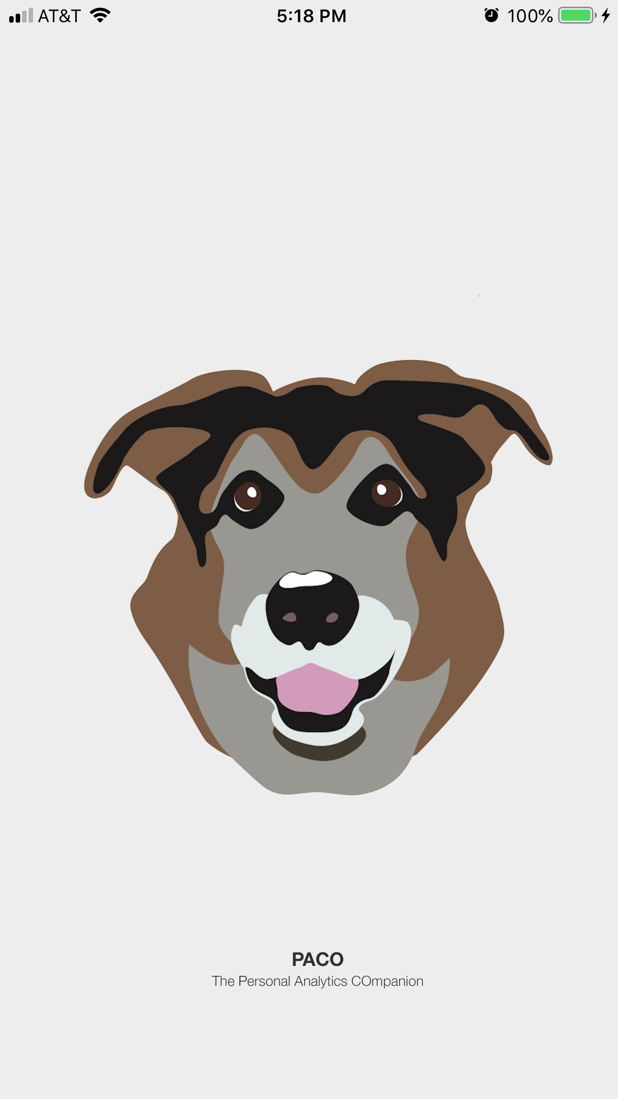

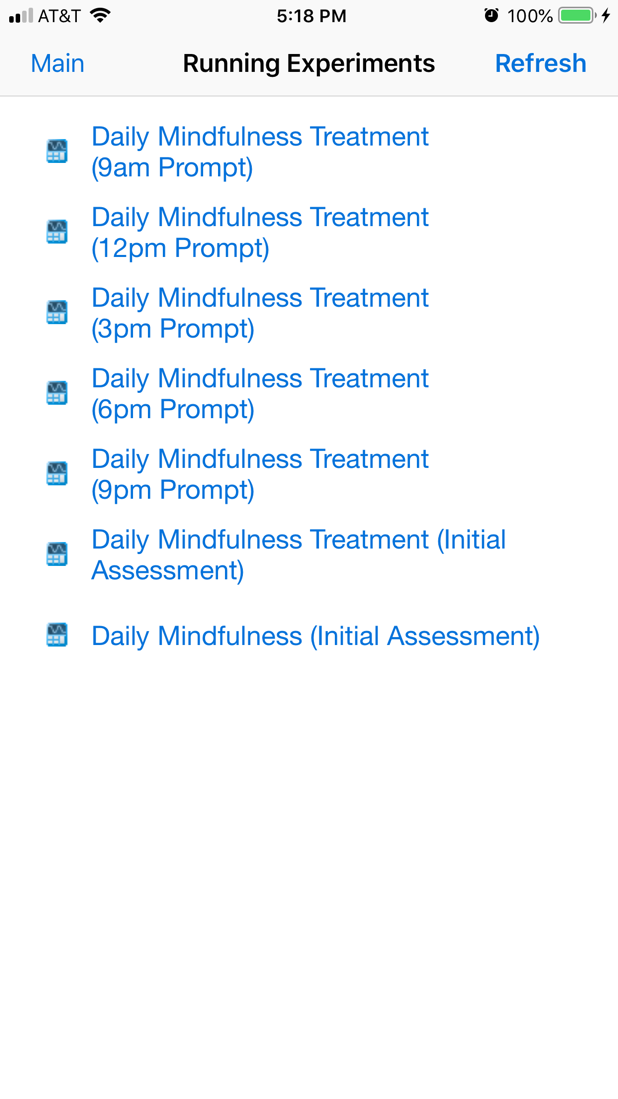


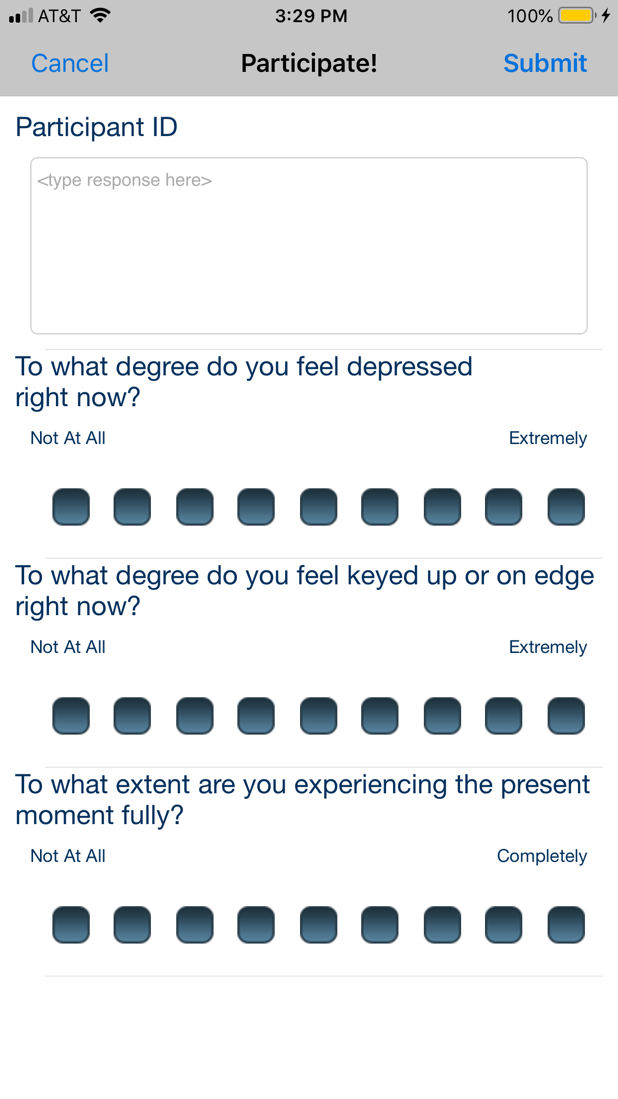

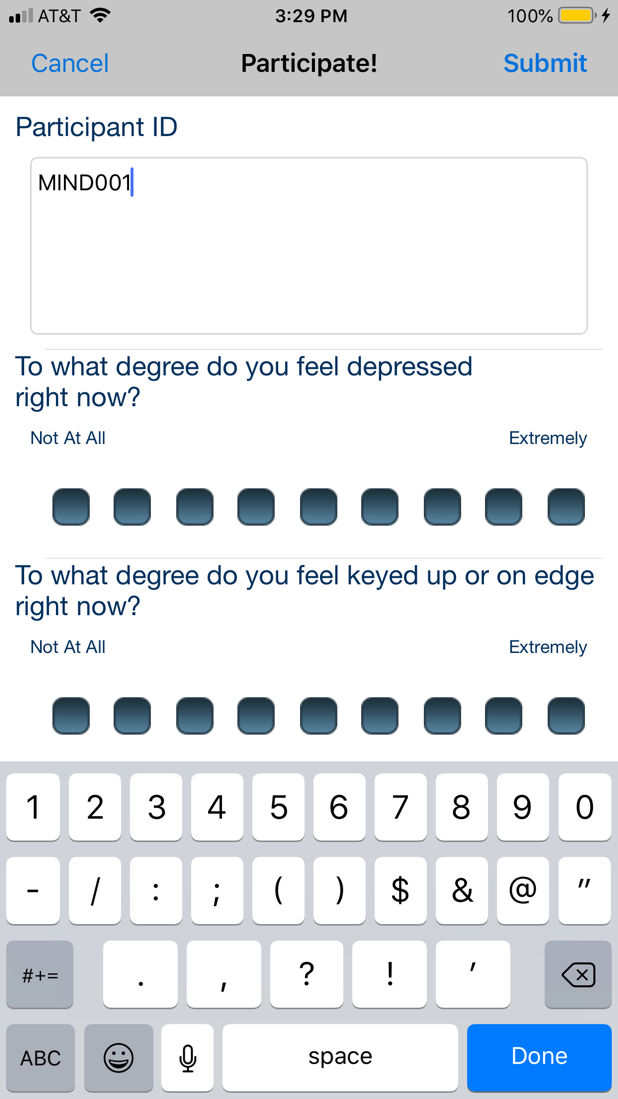


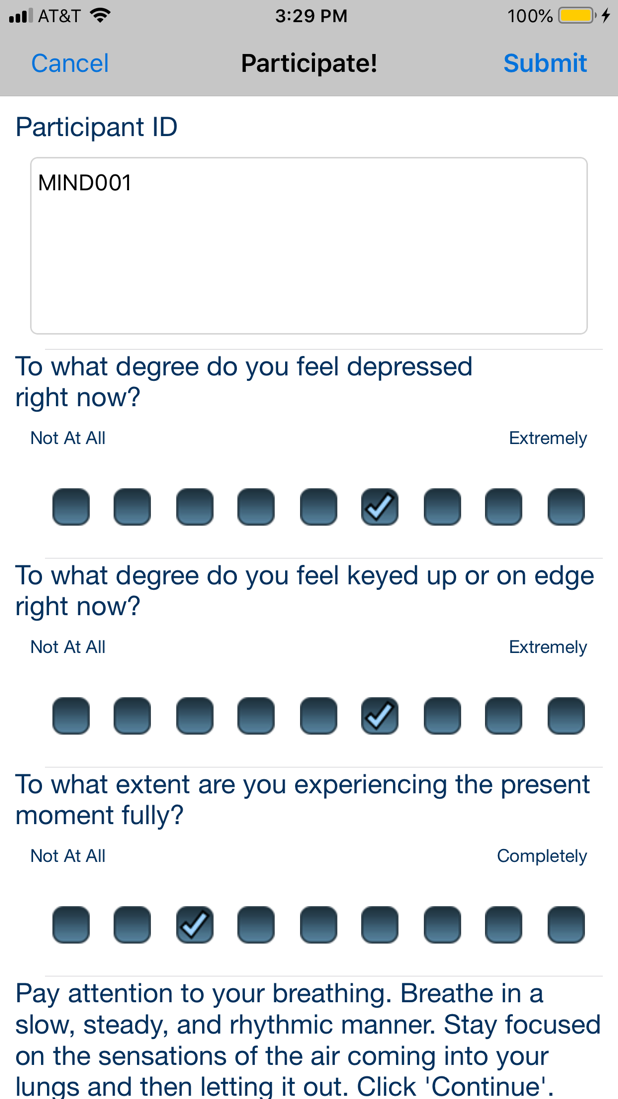

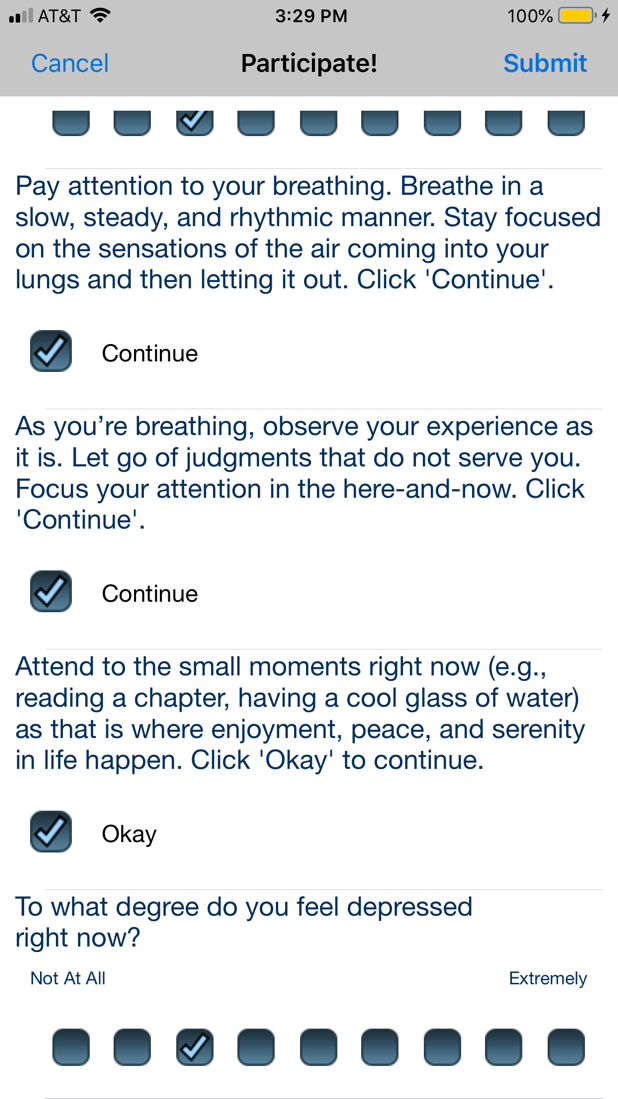


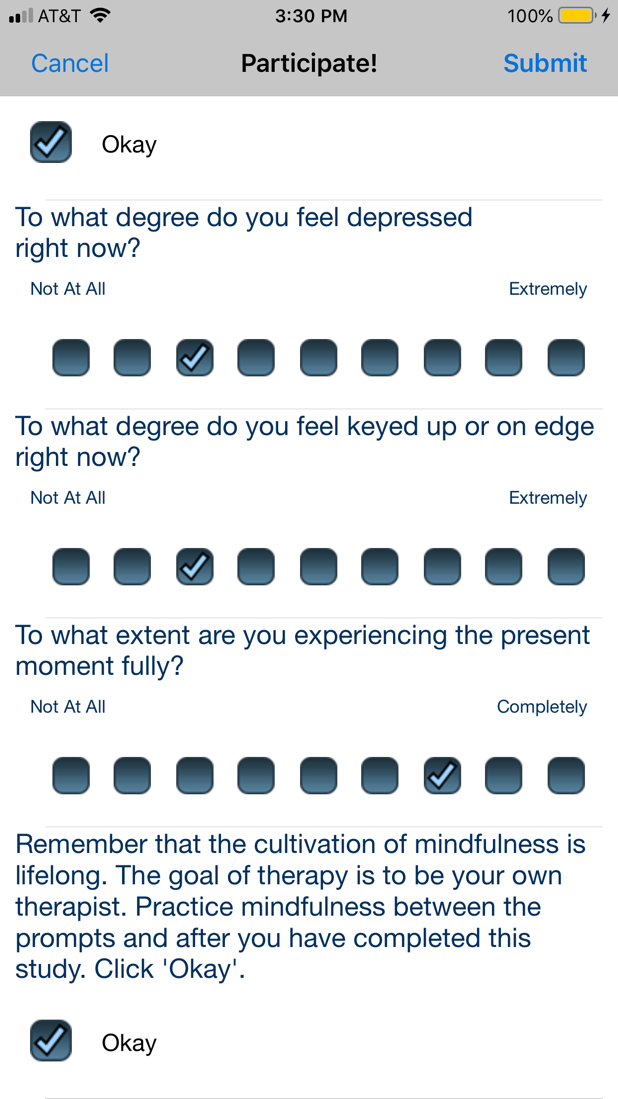

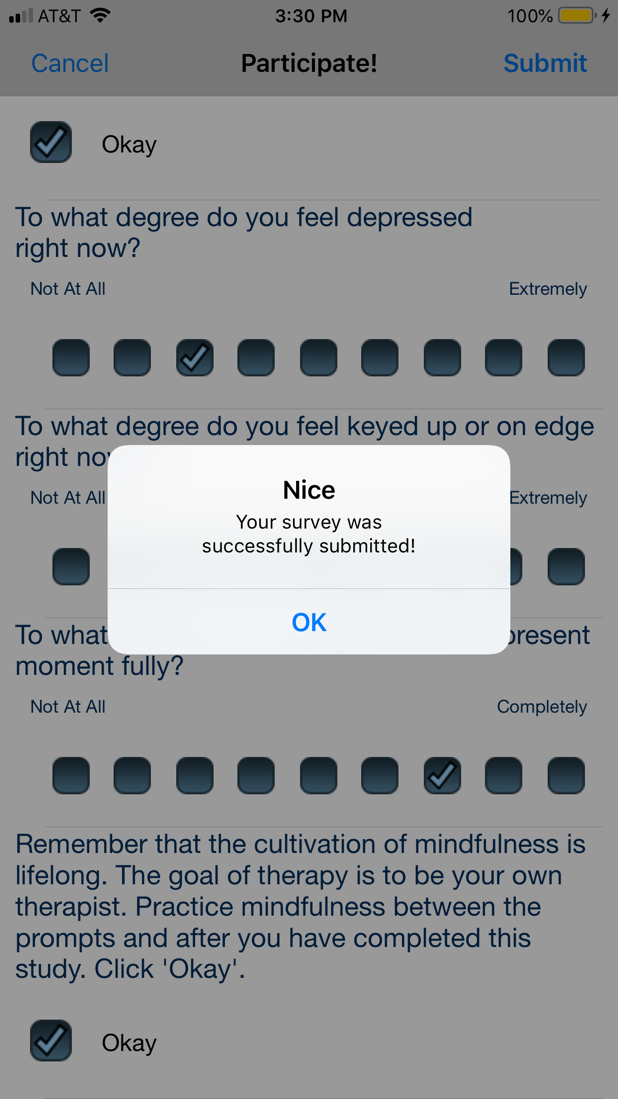


**Appendix C**

**Details of Control Arm**

In juxtaposition to the comprehensive mindfulness directives offered by the mindfulness EMI, SM individuals were given single-sentence instructions on five occasions daily (approximately at 9 a.m., 12 p.m., 3 p.m., 6 p.m., and 9 p.m.) for 14 days (Appendix C): "Notice your thoughts and how distressing they may be." We measured mindfulness, anxiety, and depression levels of participants by utilizing the same 9-level Likert scale self-reports prior to and after each SM prompt. Participants received an automated distribution of the SM informational material through Qualtrics. In contrast to the mindfulness EMI, this instructional document did not explicitly encourage participants to review its content routinely. Furthermore, SM was selected as a control based on prior theory and data (Zainal & Newman, 2023), which indicated that it was unlikely to produce notable EF enhancements but effectively served as a suitable placebo in RCTs (Lutz et al., 2022).

**Screenshots of Control Arm**

## Screenshots for self-monitoring app (SM) arm


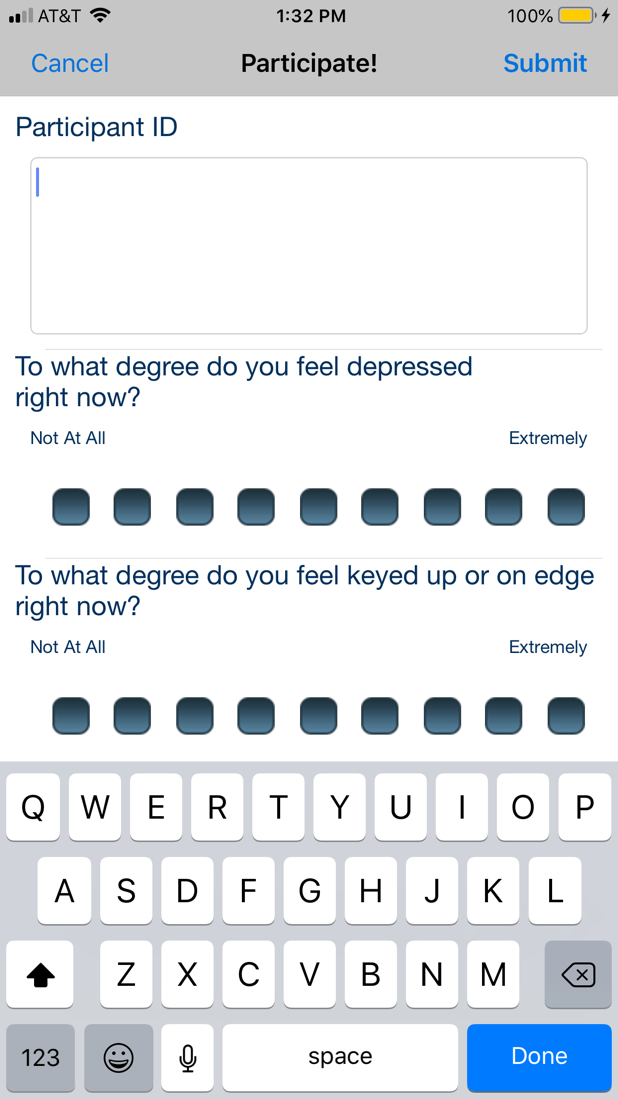

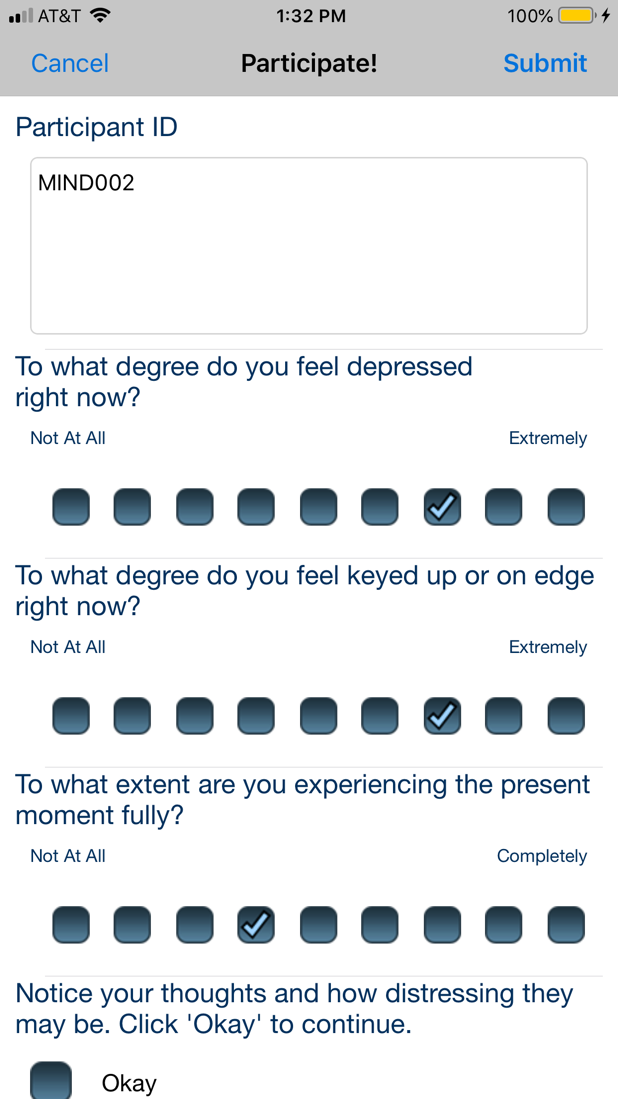


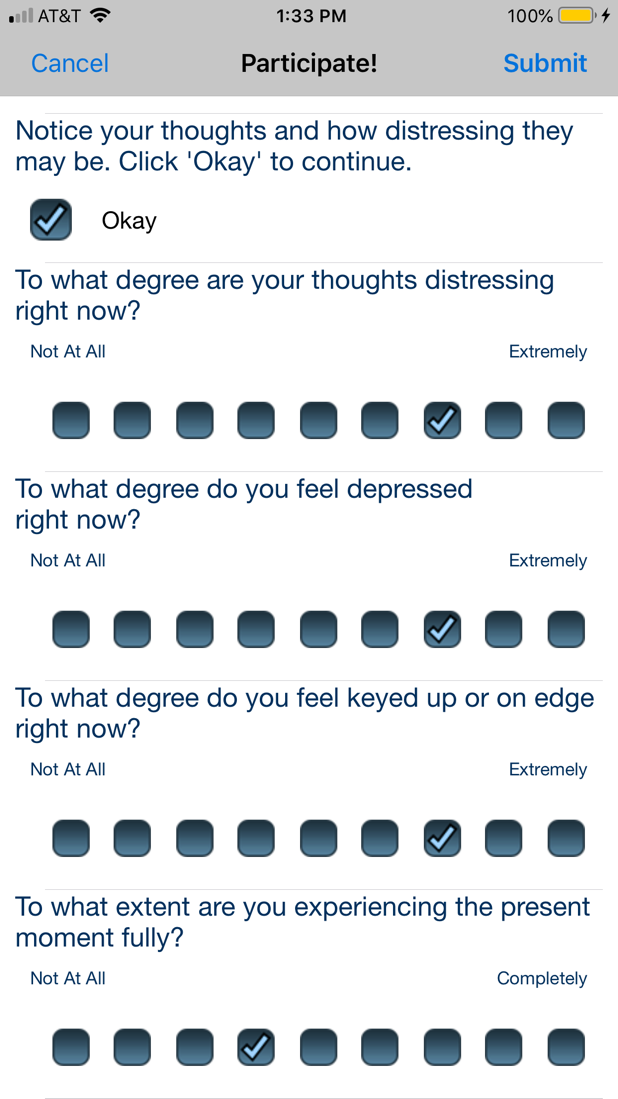

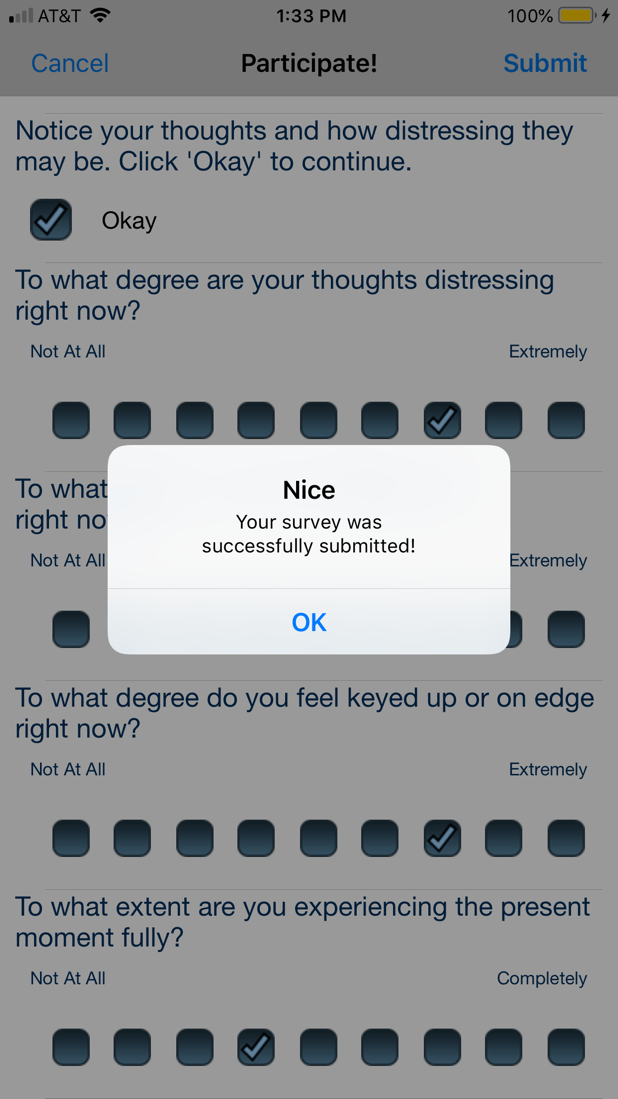


**Appendix D**

**Non-linear Mediation Models**

Table S1

*Counterfactual non-linear mediation analysis of EF domains mediating the effect of mindfulness EMI against SM on pre-1MFU GAD severity*

|  | Predicting pre-post mediator  (*a* path) | | |  | Predicting pre-1MFU GAD severity  (*b* path) | | |
| --- | --- | --- | --- | --- | --- | --- | --- |
|  | β | [LCI, UCI] | *p* |  | β | [LCI, UCI] | *p* |
| A. Inhibitory control | | | |  |  | | |
| Group x Time | -2.075^**^ | [-3.383, -0.767] | .002 |  | -7.756^***^ | [-11.176, -4.336] | .000 |
| B. Verbal fluency | | | |  |  | | |
| Group x Time | -0.991 | [-4.215, 2.233] | .547 |  | -8.063^***^ | [-11.43, -4.697] | .000 |
| C. Working memory | | | |  |  | | |
| Group x Time | 0.512^*^ | [0.014, 1.009] | .045 |  | -8.153^***^ | [-11.541, -4.765] | .000 |
| D. Set-shifting | | | |  |  | | |
| Group x Time | -2.916^*^ | [-5.133, -0.699] | .010 |  | -8.180^***^ | [-11.602, -4.758] | .000 |
| E. Attentional control | | | |  |  | | |
| Group x Time | 0.866 | [-0.898, 2.63] | .337 |  | -7.715^***^ | [-10.989, -4.44] | .000 |
| Spline regressions |  |  |  |  | *F* | *P* |  |
| Inhibitory control |  | – |  |  | 2.037 | .084 |  |
| Verbal fluency |  | – |  |  | 0.625 | .430 |  |
| Working memory |  | – |  |  | 0.054 | .817 |  |
| Set-shifting |  | – |  |  | 1.397 | .182 |  |
| Attentional control |  | – |  |  | 19.712^***^ | .000 |  |

*Note.* ^*^ *p* < .05; ^**^ *p* < .01; ^***^ *p* < .001.

β, unstandardized regression estimate; 1MFU, one-month follow-up; EF, executive functioning; EMI, ecological momentary intervention; GAD, generalized anxiety disorder; LCI, lower limit of the 95% confidence interval [CI]; UCI, upper limit of the 95% CI. The main effects (not shown in this summary table) were adjusted for within each model testing a specific mediator.

Table S2

*Counterfactual non-linear mediation analysis of EF domains mediating the effect of mindfulness EMI against SM on pre-1MFU trait repetitive thinking*

|  | Predicting pre-post mediator  (*a* path) | | |  | Predicting pre-1MFU trait repetitive thinking (*b* path) | | |
| --- | --- | --- | --- | --- | --- | --- | --- |
|  | β | [LCI, UCI] | *p* |  | β | [LCI, UCI] | *p* |
| A. Inhibitory control | | | |  |  | | |
| Group x Time | -2.075^**^ | [-3.383, -0.767] | .002 |  | -0.297^***^ | [-0.436, -0.157] | .000 |
| B. Verbal fluency | | | |  |  | | |
| Group x Time | -0.991 | [-4.215, 2.233] | .547 |  | -0.311^***^ | [-0.449, -0.174] | .000 |
| C. Working memory | | | |  |  | | |
| Group x Time | 0.512^*^ | [0.014, 1.009] | .045 |  | -0.300^***^ | [-0.438, -0.162] | .000 |
| D. Set-shifting | | | |  |  | | |
| Group x Time | -2.916^*^ | [-5.133, -0.699] | .010 |  | -0.310^***^ | [-0.45, -0.171] | .000 |
| E. Attentional control | | | |  |  | | |
| Group x Time | 0.866 | [-0.898, 2.63] | .337 |  | -0.282^***^ | [-0.408, -0.155] | .000 |
| Spline regressions |  |  |  |  | *F* | *p* |  |
| Inhibitory control |  | – |  |  | 2.082 | .081 |  |
| Verbal fluency |  | – |  |  | 1.828 | .177 |  |
| Working memory |  | – |  |  | 1.178 | .279 |  |
| Set-shifting |  | – |  |  | 1.185 | .224 |  |
| Attentional control |  | – |  |  | 59.450^***^ | .000 |  |

*Note.* ^*^ *p* < .05; ^**^ *p* < .01; ^***^ *p* < .001.

β, unstandardized regression estimate; 1MFU, one-month follow-up; EF, executive functioning; EMI, ecological momentary intervention; LCI, lower limit of the 95% confidence interval [CI]; UCI, upper limit of the 95% CI. The main effects (not shown in this summary table) were adjusted for within each model testing a specific mediator.
